# 

**Published:** 1909-05-01

**Authors:** 


### GRADUATES' MEDICAL SCHOOLS.

DIARY FOR THE WEEK ENDING MAY 3 TO MAY 8.

**MEDICAL GRADUATES' COLLEGE AND POLYCLINIC, 22 Chenies Street, W.C.**

**At 4 p.m.**
**May 3,** Dr. Willmott Evans, **Skin.**
**At 5.15 p.m.**
**May 3,** Dr. E. Cautley, **The Treatment of Broncho-Pneumonia.**
**At 4 p.m.**
**May 4,** Sir Wm. Gowers, M.D., **Medical.**
**At 5.15 p.m.**
**May 4,** Dr. George Pernet, **Feigned Eruptions.**
**At 4 p.m.**
**May 5,** Mr. James Cantlie, **Surgery.**
**At 5.15 p.m.**
**May 5,** Mr. Bland-Sutton, **The Baneful Effects of Pregnancy on Uterine Fibroids.**
**At 4 p.m.**
**May 6,** Sir Jonathan Hutchinson, **Surgery.**
**At 5.15 p.m.**
**May 6,** Dr. G. H. Savage, **The Feeble-minded and their Care.**
**At 4 p.m.**
**May 7,** Mr. Sydney Stephenson, **Eye.**

THE HOSPITAL May 1, 1909.

*Name* ..............................................................................................

*Address* ..........................................................................................

..........................................................................................................

**This Coupon must accompany manuscript or contributions intended for The Hospital.**

